# Circulating Lycopene and β-Carotene Levels Are Inversely Associated with Carotid Intima–Media Thickness: A Systematic Review and Meta-Analysis

**DOI:** 10.3390/nu18071043

**Published:** 2026-03-25

**Authors:** Iván Cavero-Redondo, Alicia Saz-Lara, Andrea Del Saz-Lara, Óscar Martínez-Cifuentes, Iris Otero-Luis, Ana González-Collado, Juan Pablo Rey-López

**Affiliations:** 1 CarVasCare Research Group, Instituto de Biomedicina/Facultad de Enfermería de Cuenca, Universidad de Castilla-La Mancha, 16001 Cuenca, Spain; ivan.cavero@uclm.es (I.C.-R.); alicia.delsaz@uclm.es (A.S.-L.); oscar.mcifuentes@uclm.es (Ó.M.-C.); iris.otero@uclm.es (I.O.-L.); ana.gonzalezcollado@uclm.es (A.G.-C.); 2 Facultad de Ciencias de la Salud, Universidad Tecnológica Atlántico Mediterráneo (UTAMED), C. de Marie Curie, 1, Campanillas, 29590 Málaga, Spain; 3 Faculty of Health Sciences, Universidad Internacional de Valencia (VIU), 46002 Valencia, Spain; jprey@universidadviu.com

**Keywords:** carotenoids, lycopene, β-carotene, carotid intima-media thickness, atherosclerosis, cardiovascular risk

## Abstract

**Background**: Carotid intima-media thickness (IMT) is a well-established surrogate marker of subclinical atherosclerosis and a predictor of cardiovascular risk. Carotenoids, particularly lycopene and β-carotene, have been proposed as protective antioxidants against vascular damage, but evidence from population-based studies is inconsistent. **Objective**: We aim to perform a systematic review and meta-analysis of the associations between circulating levels of lycopene and β-carotene and carotid IMT in the general adult population, including potential sex-specific effects. **Methods**: A systematic search was conducted in PubMed, Scopus, and Web of Science up to March 2025, following PRISMA guidelines (PROSPERO registration: CRD420251003810). Observational and experimental studies reporting cross-sectional associations between plasma carotenoids and IMT were included. Pooled odds ratios (ORs) with 95% confidence intervals (CIs) were calculated via random effects models. Subgroup and meta-regression analyses explored potential modifiers, including sex and cardiovascular risk factors. **Results**: Thirteen studies (*n* = 9131; mean age 46.4–71.6 years) met the inclusion criteria, eight of which were eligible for meta-analysis. High circulating lycopene levels were significantly associated with low IMT (pooled OR = 0.70; 95% CI: 0.59–0.84; *I*^2^ = 65.7%). The association was stronger in men (OR = 0.62; 95% CI: 0.45–0.84) than in women (OR = 0.74; 95% CI: 0.58–0.95). In contrast, β-carotene was only marginally associated with IMT (pooled OR = 0.96; 95% CI: 0.92–0.99; *I*^2^ = 72.6%). Meta-regression suggested that systolic blood pressure modified the lycopene-IMT relationship, whereas body mass index and low-density lipoprotein cholesterol influenced the β-carotene-IMT association. No evidence of publication bias was found. **Conclusions**: Increased serum lycopene concentrations, and to a lesser extent β-carotene concentrations, are inversely associated with carotid IMT, suggesting a protective role of lycopene in vascular health. The effect appears more pronounced in men, highlighting potential sex-specific differences in carotenoid metabolism and cardiovascular risk modulation.

## 1. Introduction

Atherosclerosis is a leading cause of cardiovascular diseases (CVDs) globally and remains a significant public health concern because of its high morbidity and mortality rates [1]. This is a progressive condition characterized by the accumulation of lipids and fibrous elements in arterial walls, which contributes to major complications such as myocardial infarction, stroke, and peripheral arterial disease [2]. Understanding the underlying factors and mechanisms driving atherosclerosis is crucial for developing preventive and therapeutic strategies [3]. This disease is initiated by endothelial dysfunction, which promotes subendothelial retention and the oxidation of low-density lipoprotein (LDL) particles, followed by monocyte infiltration and foam cell formation. These early lesions evolve into fatty streaks and plaques through smooth muscle cell migration and extracellular matrix deposition, processes that are amplified by chronic inflammation. Major risk factors such as dyslipidemia, hypertension, diabetes, smoking, obesity, and genetic predisposition accelerate this cascade, highlighting the multifactorial nature of atherogenesis [4].

The assessment of atherosclerosis has improved significantly, with carotid intima-media thickness (IMT) being among the most widely used and validated noninvasive markers [5]. IMT measurement, obtained via high-resolution ultrasonography, serves as a marker of subclinical atherosclerosis, providing valuable insights into the early stages of vascular changes and contributing to the prediction of future cardiovascular events [6]. Its utility lies in its ability to detect subtle changes in arterial walls, offering a window into the progression of atherosclerosis before clinical symptoms appear [7].

Among the different factors influencing IMT, dietary antioxidants have been highlighted as key players in maintaining vascular health [8,9]. Antioxidants play pivotal roles in neutralizing oxidative stress, which is a key contributor to endothelial dysfunction and the initiation of atherogenesis [10]. Among the variety of dietary antioxidants, carotenoids, which are natural pigments in fruits and vegetables, have attracted attention because of their potential protective effects against atherosclerosis [11,12,13].

Carotenoids, such as β-carotene and lycopene, exhibit potent antioxidant properties that may mitigate the oxidative damage associated with the development of atherosclerosis [14]. Emerging evidence suggests an inverse association between serum levels of these carotenoids and IMT, suggesting a possible role in maintaining vascular health [12,15]. Importantly, several studies have indicated that sex may modify this relationship. For example, in the Kuopio Ischaemic Heart Disease Risk Factor Study, higher serum lycopene concentrations were inversely associated with carotid IMT progression in men, whereas the effect was less pronounced in women, suggesting sex-specific modulation of antioxidant metabolism and vascular outcomes [16,17]. Moreover, women tend to have higher circulating carotenoid levels than men do, potentially because of differences in lipid metabolism, hormonal status, and adipose tissue distribution, which may further influence vascular responses.

Despite growing interest in the role of circulating carotenoids as potential biomarkers of vascular health, the available evidence remains inconsistent, with studies reporting heterogeneous findings across different populations and study designs. In this context, a systematic review and meta-analysis is warranted to provide a comprehensive synthesis of the literature, quantify the magnitude of the association between carotenoids and carotid intima–media thickness, and improve comparability across studies using different methodologies. Furthermore, such an approach may help identify potential sources of variability, including sex differences and the influence of cardiovascular risk factors, thereby contributing to a more nuanced understanding of the relationship between carotenoids and subclinical atherosclerosis. On the basis of these considerations, the present study aimed to evaluate the associations between serum β-carotene and lycopene levels and carotid IMT. In addition, this study aims to explore possible sex-specific differences in this relationship to provide a more complete understanding of how these carotenoids contribute to vascular health and the progression of atherosclerosis.

## 2. Materials and Methods

This systematic review and meta-analysis was performed in compliance with the Preferred Reporting Items for Systematic Reviews and Meta-analyses (PRISMA) guidelines [18] and was performed following the recommendations of the Cochrane Collaboration Handbook [19]. Reporting was further aligned with the PRISMA 2020 checklist to ensure transparency and completeness (Table S1). This study was registered in PROSPERO (registration number: CRD420251003810).

### 2.1. Search Strategy

A systematic search was conducted through three databases, PubMed, Scopus, and Web of Science, from their inception to 30 March 2025. These databases were selected because they comprehensively cover biomedical, clinical, and nutritional research and are widely recognized as core sources for systematic reviews in health sciences.

To perform the search in the Pubmed, Scopus and Web of Science databases, a comprehensive strategy was developed using free-text terms combined with Boolean operators (AND, OR), structured according to the PICO framework (population, intervention, comparator, outcome) [20]. The search strategy was as follows: (“antioxidant lycopene” OR lycopene OR “plasma lycopene” OR “serum lycopene” OR “plasma carotenoids” OR “serum carotenoid” OR carotenoids OR “antioxidant vitamins” OR “plasma antioxidants” OR antioxidants OR β-carotene OR α-carotene OR β-cryptoxanthin OR lutein OR zeaxanthin) AND (“carotid intima-media thickness” OR “intima-media thickness” OR CIMT OR IMT) AND (atherosclerosis OR “carotid artery wall” OR “carotid atherosclerosis” OR “carotid atherosclerotic disease” OR “carotid arteries”). The detailed search strategy applied in PubMed is provided in Table S2. In addition, the reference lists of relevant systematic reviews, meta-analyses, and included studies were manually screened to identify any additional eligible articles.

### 2.2. Eligibility Criteria

The inclusion criteria were as follows: (i) population: adult population (≥18 years), including both apparently healthy individuals and individuals with cardiometabolic risk factors or established cardiovascular disease; (ii) exposure to antioxidants, including lycopene and β-carotene; (iii) outcome: IMT; (iv) study design: cross-sectional data from longitudinal studies (cohort and experimental studies); and (v) no language restrictions. In view of the limited availability of studies specifically examining the association between circulating carotenoids and IMT, a broad inclusion of study designs was considered appropriate to provide a comprehensive overview of the existing evidence. Accordingly, the unit of synthesis was cross-sectional association data between circulating carotenoids and IMT, regardless of the parent study design (e.g., cohort, case–control, randomized controlled trials (RCTs), or cross-sectional studies). This approach allowed the inclusion of diverse study designs while maintaining comparability by restricting the quantitative synthesis to cross-sectional associations.

We excluded the following: (i) review articles, case reports, or editorials; (ii) studies that did not include cross-sectional data on the association between antioxidants and IMT; and (iii) studies that included other interventions, such as diet or exercise.

### 2.3. Data Extraction

A structured table was developed to summarize the key characteristics of the included studies. The extracted information included (1) study identification (first author and year of publication); (2) country of data collection; (3) study design, including cross-sectional data derived from cohort studies, case–control studies, randomized controlled trials (RCTs), or purely cross-sectional studies; (4) population characteristics, such as sample size, proportion of female participants, mean age, and prevalence of comorbid conditions; (5) exposure variables, including the type of antioxidants assessed (lycopene and β-carotene) and their baseline circulating levels; and (6) outcome measures, specifically carotid intima–media thickness (IMT), including measurement methods and baseline values.

To avoid potential nonindependence of observations, studies originating from the same research groups or cohorts were carefully examined to identify possible overlap in participant populations. Not all the studies included in the systematic review were incorporated into the quantitative meta-analysis. When multiple publications were derived from the same or closely related samples, only one dataset was included in each pooled analysis. Furthermore, separate meta-analyses were conducted according to the type of antioxidant (lycopene or β-carotene), ensuring that the same study population was not included more than once within a given pooled estimate. This process was carefully reviewed and verified during data extraction.

In addition, data on effect estimates were extracted when available, including correlation coefficients (r), regression coefficients (β), odds ratios (ORs), and mean differences. Only cross-sectional or baseline associations between circulating carotenoids and IMT were considered for the quantitative synthesis. When studies reported multiple effect estimates (e.g., across different exposure categories, subgroups, or analytical models), a single estimate was selected to avoid double counting. Priority was given to estimates derived from the overall study population and those based on the most comparable exposure definitions across studies. When necessary, selection decisions were made by consensus between the reviewers. The set of covariates included in the adjustment models (e.g., age, sex, BMI, smoking status, blood pressure, and lipid profile) was also recorded when available. This information was used to assess the degree of comparability across studies and to inform the interpretation of pooled estimates.

Given the heterogeneity in reporting formats, effect sizes were harmonized into a common metric (OR) to facilitate comparability and interpretation. Correlation coefficients (r) were first transformed using Fisher’s z transformation and subsequently converted into ORs using established statistical relationships. Regression coefficients (β) and mean differences between exposure categories were standardized and transformed into ORs using conventional effect size conversion methods. When direct analytical conversion was not feasible because of incomplete reporting or incompatible metrics, effect sizes were estimated using the Campbell Collaboration effect size calculator. Only studies with effect estimates that could be reliably transformed into ORs were included in the quantitative meta-analysis, whereas the remaining studies were included in the qualitative synthesis. Detailed information on the original effect measures and their corresponding transformations is provided in the Supplementary Materials (Tables S3 and S4).

When multiple effect estimates were available, priority was given to those derived from the most fully adjusted models to minimize confounding. In addition, predefined criteria were applied to select a single estimate per study, prioritizing overall population estimates and the most comparable exposure definitions across studies.

### 2.4. Quality Assessment

The risk of bias was assessed using the National Heart, Lung, and Blood Institute (NHLBI) quality assessment tools, which were selected according to the study design. Specifically, the tool for observational cohort and cross-sectional studies was applied to cross-sectional and prospective studies (14 items) [21], the tool for case–control studies was used for retrospective designs (12 items) [22], and the tool for controlled intervention studies was employed for randomized trials (14 items) [23]. On the basis of these assessments, studies were classified as “good” (low risk of bias, most criteria met), “fair” (moderate risk of bias, some criteria met), or “poor” (high risk of bias, few criteria met).

The literature search, study selection, data extraction, and quality assessment processes were conducted independently by two reviewers (I.C.-R. and A.S.-L.). Any discrepancies were resolved through discussion and consensus or, when necessary, by consultation with a third reviewer (J.P.R.-L.).

### 2.5. Statistical Analysis

Random-effects meta-analyses were conducted using the Hartung–Knapp–Sidik–Jonkman (HKSJ) method [24], which provides more robust estimates of confidence intervals, particularly in the presence of a small number of studies and substantial heterogeneity. Pooled OR estimates and their 95% confidence intervals (95% CIs) were calculated to assess the associations between circulating carotenoids (lycopene and β-carotene) and IMT in heterogeneous adult populations. All pooled estimates were based on harmonized ORs derived from the transformation of different effect measures reported across studies (Tables S3 and S4). Heterogeneity was assessed via the *I*^2^ statistic, which ranged from 0 to 100%. According to the *I*^2^ values, heterogeneity was considered not important (0–30%), moderate (30–60%), substantial (60–75%), or considerable (75–100%) [25]. The corresponding *p* values were also considered.

Although IMT is a continuous outcome, ORs were used as a common effect size metric to harmonize heterogeneous measures across studies and facilitate interpretation of the strength of association. This approach allowed the integration of results derived from different analytical frameworks (e.g., correlations, linear regression coefficients, and categorical comparisons) into a unified metric, enabling quantitative synthesis while acknowledging the inherent approximation involved in such transformations.

Only studies with effect estimates that could be reliably transformed into a common OR metric were included in the quantitative synthesis. When heterogeneity in effect measures or reporting precluded reliable transformation or direct pooling, the findings were summarized narratively. Accordingly, inclusion in the quantitative meta-analysis required that studies reported cross-sectional or baseline associations and provided effect estimates that could be harmonized into a common OR metric. This ensured methodological consistency across pooled analyses despite differences in parent study design.

Sensitivity analyses were conducted by sequentially removing individual studies (leave-one-out approach) to evaluate the stability of the pooled estimates for each antioxidant (lycopene and β-carotene). Subgroup analyses were performed according to sex (male and female). Random-effects meta-regression analyses were additionally carried out to explore whether study-level variables, including mean age, body mass index (BMI), systolic blood pressure (SBP), diastolic blood pressure (DBP), total cholesterol, high-density lipoprotein (HDL) cholesterol, low-density lipoprotein (LDL) cholesterol, triglyceride levels, and smoking status, were associated with variations in the observed effect sizes. These meta-regression analyses were considered exploratory and aimed at identifying potential sources of heterogeneity. Publication bias was assessed using Egger’s regression asymmetry test for each antioxidant [26], with a significance threshold set at *p* < 0.10.

All the statistical analyses were performed using STATA SE software (version 19; StataCorp, College Station, TX, USA).

## 3. Results

### 3.1. Baseline Characteristics

**Figure 1:**
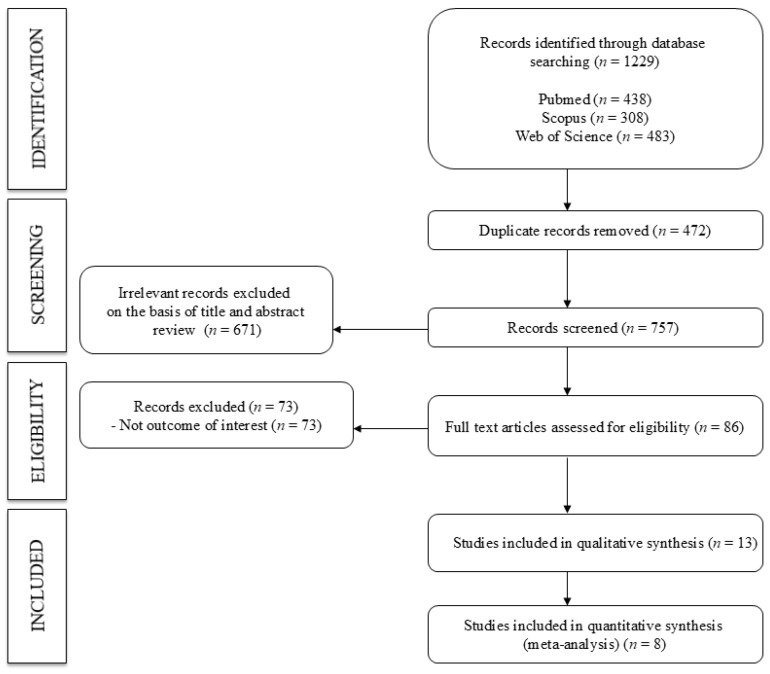
Flowchart of studies that examined the association between lycopene or beta carotene and IMT (PRISMA-2020).

In total, 13 studies [27,28,29,30,31,32,33,34,35,36,37,38,39] were included in the systematic review, and 8 studies were included in the meta-analysis [28,29,30,31,35,36,37,39] (Figure 1). Among the included studies, seven were cohort studies [28,29,32,33,34,35,36], two were RCTs [27,31], two were case-control studies [31,39], and two were cross-sectional studies [34,40]; these studies were performed in different countries: four in Finland [27,31,32,35], three in China [37,38,39], two in Italy [30,36] and the USA [33,34], and one each in the United Kingdom [28] and Australia [29]. The studies were published between 2000 and 2018 and included a total of 9131 subjects (aged 46.4–71.6 years) with different pathologies, such as hypertension, diabetes, or hyperlipidemia. Eleven studies included data on lycopene levels [27,29,30,31,32,33,35,36,37,38,39], and ten studies included data on β-carotene levels [28,29,30,32,33,34,35,37,38,39]. Finally, different methods were used to measure the IMT, with the most commonly used methods being Biosound Phase 2 in three studies [27,31,32], Acuson Sequoia C512 in three studies [33,34,36], and Aloka Prosound α-10 in two studies [38,39]. The characteristics of the studies included in the systematic review are shown in Table 1 and Table S5.

Table S6 summarizes the covariates included in the adjustment models of the studies contributing to the meta-analysis of β-carotene, lycopene and IMT. Overall, there was substantial variability in adjustment strategies across studies, ranging from fully adjusted multivariable models including key cardiovascular risk factors and lifestyle variables [28,29,39] to partially adjusted or unadjusted analyses. Notably, several studies reported crude associations without adjustment for potential confounders, whereas others included only limited covariates such as age and sex or lipid parameters. This heterogeneity in covariate adjustment may have influenced the magnitude of the observed associations and should be considered when the pooled estimates are interpreted.

### 3.2. Quality Assessment

**Figure 2:**
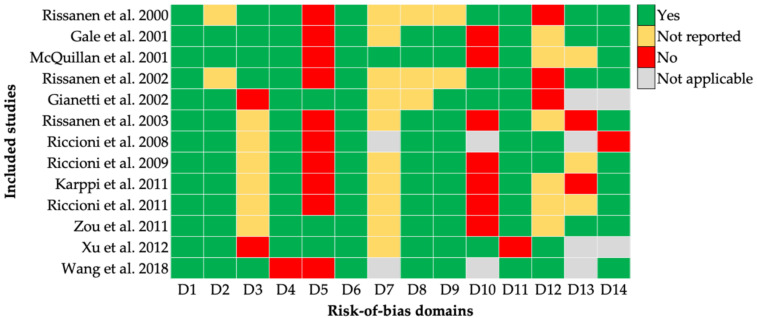
Domain-level risk-of-bias assessment across included studies. The risk of bias was assessed using the National Heart, Lung, and Blood Institute quality assessment tools according to the study design. Each study was evaluated across multiple domains. Green indicates a low risk of bias (“Yes”), yellow indicates an unclear risk (“Not reported”), red indicates a high risk of bias (“No”), and gray indicates not applicable. Detailed assessments are provided in Supplementary Tables S4–S6 [27,28,29,30,31,32,33,34,35,36,37,38,39].

The overall risk of bias for studies examining the association between antioxidants and IMT in the general population was rated as good in 53.8% and fair in 46.2% of the included studies (Tables S7–S9). A visual summary of the domain-level risk-of-bias assessment is provided in Figure 2.

Detailed domain-level assessments (Tables S4–S6) indicate that although most studies adequately reported objectives, population characteristics, and outcome measurements, several methodological limitations were consistently observed. The main sources of potential bias included the lack of sample size justification, limited information on participant blinding, and insufficient reporting of repeated exposure assessments over time.

In addition, several studies reported incomplete reporting in domains related to exposure measurement and confounding control, which may introduce information bias and residual confounding. Cross-sectional designs were also unable to address domains related to follow-up, which inherently limits causal interpretation. These aspects should be considered when the pooled estimates are interpreted.

### 3.3. Associations Between Antioxidants (Lycopene and β-Carotene) and the Thickness of the Intima Media

**Figure 3:**
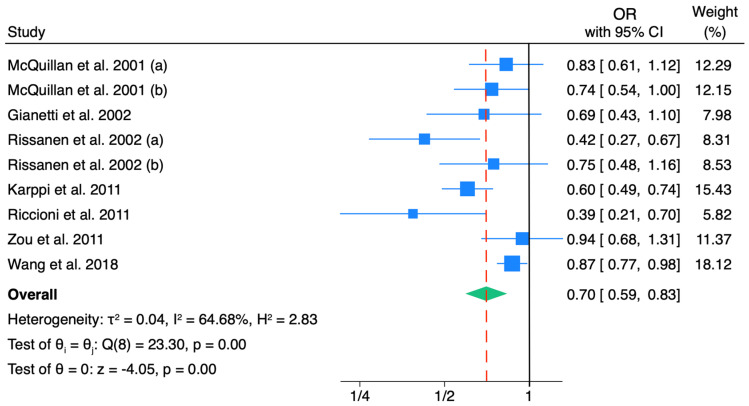
Forest plot including the association between lycopene levels and intima–media thickness. The subsamples included in (a) and (b) refer to two different populations included in the manuscript [29,30,31,35,36,37,39].

The pooled estimates of the OR between lycopene and IMT in the general population were 0.70 (95% CI: 0.59, 0.83), with substantial heterogeneity (*I*^2^: 64.7%) (Figure 3). This level of heterogeneity suggests moderate variability in effect sizes across studies, likely reflecting differences in population characteristics, exposure assessment methods, and covariate adjustment strategies.

**Figure 4:**
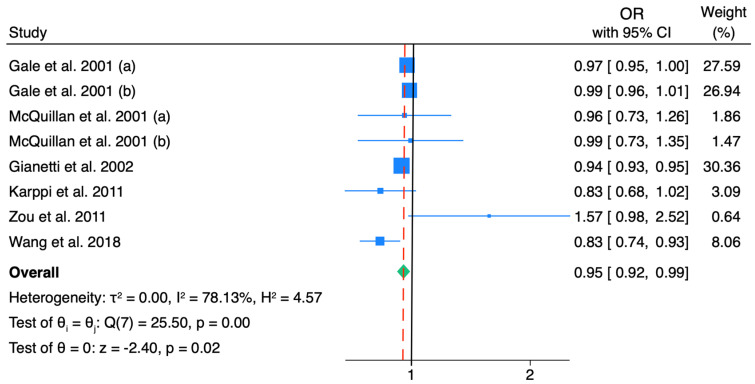
Forest plot including the association between β-carotene levels and IMT. The subsamples included in (a) and (b) refer to two different populations included in the manuscript [28,29,30,35,37,39].

The pooled ORs for β-carotene and IMT in the general population were 0.95 (95% CI: 0.92, 0.99), with substantial heterogeneity (*I*^2^: 78.1%) (Figure 4). The higher heterogeneity observed in this analysis indicates greater inconsistency across studies, which may be related to variability in β-carotene bioavailability, study populations, and methods.

### 3.4. Sensitivity Analysis, Subgroup Analysis, Meta-Regression Models, and Publication Bias

The pooled OR estimates for the associations between antioxidants (lycopene and β-carotene) and IMT were not significantly modified (in magnitude or direction) when data from individual studies were removed one at a time from the analysis. The observed changes in pooled estimates were minimal, indicating that no single study disproportionately influenced the overall results and supporting the robustness of the meta-analytic findings.

For subgroup analysis according to sex (male or female), the pooled OR estimate for the association between lycopene and IMT was 0.61 (95% CI: 0.44, 0.86) for males and 0.74 (95% CI: 0.58, 0.95) for females. Although the association appeared slightly stronger in males, the direction of the effect was consistent across sexes and the difference in magnitude was moderate, suggesting that sex may influence the strength but not the overall pattern of the association. When the association between β-carotene and IMT according to sex was analyzed, the pooled OR estimate was 0.95 (95% CI: 0.88, 1.03) for males and 0.99 (95% CI: 0.96; 1.01) for females (Table S10), with minimal differences between subgroups, indicating a lack of meaningful effect modification by sex.

Random effects meta-regression models revealed that SBP could influence the pooled OR estimate for the association between lycopene and IMT (*p* = 0.024), and BMI and LDL cholesterol could influence the pooled OR estimate for the association between β-carotene and IMT (*p* = 0.043 and *p* = 0.026, respectively) (Table S11).

Egger’s test did not reveal statistical evidence of small-scale effects for lycopene (*p* = 0.101) (Figure S1) or β-carotene (*p* = 0.681) (Figure S2). However, given the limited number of included studies, this analysis is underpowered and should be interpreted with caution.

## 4. Discussion

The present systematic review and meta-analysis revealed that higher circulating levels of lycopene and, to a lesser extent, β-carotene are associated with lower carotid IMT in heterogeneous adult populations, including individuals with and without cardiometabolic conditions. These findings indicate an inverse association between circulating carotenoid levels and IMT; however, they should not be interpreted as evidence of a causal relationship. Given the predominantly cross-sectional nature of the included studies, reverse causation and residual confounding cannot be excluded. From a clinical standpoint, these findings support the potential relevance of carotenoid-rich dietary patterns in relation to vascular health, although causal inferences remain limited. In addition, it should be noted that studies with a higher risk of residual confounding or incomplete adjustment for cardiovascular risk factors tended to report stronger associations, suggesting that part of the observed effect may be influenced by unmeasured or inadequately controlled variables.

Our results concerning lycopene align with those of previous systematic reviews, indicating a protective role of this antioxidant against cardiovascular diseases [40,41,42,43]. Specifically, the inverse association between lycopene levels and IMT is consistent with earlier evidence highlighting the role of lycopene in reducing arterial stiffness and oxidative stress [44,45,46,47]. In contrast, the marginal effect of β-carotene observed in our study reflects the inconsistent findings reported in earlier reviews, possibly owing to its variable bioavailability and differences in population characteristics across studies [48]. Furthermore, heterogeneity in exposure assessment methods (e.g., single vs. repeated measurements, serum vs. plasma concentrations) and variability in IMT measurement protocols across studies may partly explain the observed differences in effect sizes. Measurement heterogeneity may have introduced additional variability, potentially attenuating or inflating the pooled estimates. Finally, the transformation of different effect measures (e.g., r, β coefficients, and group comparisons) into a common OR metric, although necessary for pooling, may have introduced additional approximation and contributed to residual heterogeneity. Therefore, pooled estimates should be interpreted as approximate indicators of association rather than precise effect sizes. The substantial heterogeneity observed in the main analyses (*I*^2^ = 64.7% for lycopene and 78.1% for β-carotene) warrants cautious interpretation of the pooled estimates. Although subgroup and meta-regression analyses were conducted to explore potential sources of heterogeneity, the relatively small number of studies may have limited their statistical power. Therefore, these analyses should be considered exploratory, and the identified associations should be interpreted with caution.

Beyond measurement variability, the observed heterogeneity can be attributed to several key sources, including population differences, methodological variations in IMT assessment, and differences in carotenoid measurement. First, population differences across studies, including age distribution, cardiovascular risk profiles, geographic location, and baseline nutritional status, may have influenced both circulating carotenoid levels and IMT values. Second, methodological heterogeneity was evident in the study design (cross-sectional vs. cohort-derived data vs. RCT-derived data), exposure assessment (single measurements vs. repeated measures), and analytical strategies, including differences in covariate adjustment. Third, variability in biomarker assessment, such as the use of serum versus plasma carotenoid concentrations and differences in laboratory techniques, may have contributed to inconsistencies across studies. Despite this heterogeneity, pooling of results was considered appropriate for providing an overall estimate of the association between circulating carotenoids and IMT, given the shared biological rationale and comparable direction of effect across most studies. However, the summary estimates should be interpreted with caution, as they represent an average effect across diverse study contexts rather than a uniform association. The observed heterogeneity underscores the need for standardized methodologies in future research to improve comparability and strengthen causal inference. Taken together, these sources of heterogeneity highlight that the observed associations are context-dependent and should not be interpreted as uniform effects across populations or study settings.

Several prior studies, including those by Rissanen et al. (2000, 2002, 2003) [27,31,32] and Riccioni et al. (2008, 2011) [33,36], have demonstrated an inverse relationship between serum lycopene levels and carotid IMT, which further confirms our findings. The powerful antioxidant activity of lycopene is likely fundamental to this relationship. By scavenging reactive oxygen species (ROS), lycopene reduces the oxidative modification of LDL cholesterol, a key driver of foam cell formation and atherosclerotic plaque development [49]. Additionally, lycopene improves endothelial function by increasing nitric oxide bioavailability and reducing proinflammatory cytokine expression, thereby contributing to decreased vascular remodeling and arterial thickening [50].

The association between β-carotene and IMT in our study was weak and weakly significant. This is consistent with earlier findings from McQuillan et al. (2001) [29] and Karppi et al. (2011) [35], who reported heterogeneous results. The physiological basis may lie in β-carotene’s less efficient quenching of singlet oxygen than lycopene and its conversion into vitamin A [51], which might modulate lipid metabolism and inflammatory pathways only indirectly [52]. Moreover, β-carotene is more susceptible to degradation under oxidative stress, potentially limiting its protective effects in individuals with elevated atherosclerotic burden [53].

From a clinical and epidemiological perspective, these findings suggest that the association between circulating carotenoids and vascular health may differ in magnitude and relevance depending on the specific compound. While the inverse association observed for lycopene may have potential implications at the population level, particularly in the context of dietary patterns rich in fruits and vegetables, the relatively modest effect size observed for β-carotene is unlikely to be clinically meaningful on its own. Instead, β-carotene may act as part of a broader antioxidant profile rather than as an independent determinant of vascular health. These results underscore the importance of considering whole dietary patterns rather than isolated nutrients when evaluating cardiovascular risk.

Our subgroup analysis revealed a stronger inverse association between lycopene levels and IMT in males than in females, whereas the β-carotene–IMT association remained weak in both sexes. This sex-based difference may be explained by hormonal modulation of antioxidant metabolism; for example, estrogens exert independent vasoprotective effects through antioxidant pathways, potentially masking the impact of exogenous antioxidants in women [54,55]. Men who lack oestrogens may benefit more from dietary antioxidants in terms of vascular protection [56]. Furthermore, sex-specific differences in carotenoid metabolism may explain this variability. In a study of β-carotene supplementation, BALB/c male mice presented increased hepatic expression of carotenoid-cleaving enzymes (Bco1/Bco2), increased retinol mobilization (Rbp4/Stra6l), reduced serum and hepatic lipids, and decreased visceral adiposity, accompanied by the regulation of estrogen receptors. These findings suggest that differences in carotenoid conversion and mobilization, possibly modulated by sex hormones, contribute to sex-dependent responses [57].

Meta-regression analyses suggested that SBP may influence the association between lycopene and IMT [58], whereas BMI and LDL cholesterol may play a role in the β-carotene–IMT relationship [59]. However, these findings should be interpreted with caution, as the limited number of studies reduces the statistical power and increases the risk of false-positive results. Therefore, these analyses should be considered exploratory and hypothesis-generating rather than indicative of definitive effect modification. Future studies with larger sample sizes and more consistent reporting of covariates are needed to confirm these potential associations. Importantly, the covariates included in the adjustment models varied across studies, and not all the studies controlled for key cardiovascular risk factors. This variability in adjustment strategies may have contributed to differences in effect estimates and should be considered when the pooled results are interpreted.

This study has several limitations. First, the cross-sectional design of most included studies prevents us from inferring causality. Second, heterogeneity in IMT measurement methods and population characteristics may affect generalizability. In particular, the inclusion of populations with varying cardiometabolic profiles (including individuals with established cardiovascular disease or risk factors) may have influenced the observed associations, as baseline cardiovascular risk differs substantially across studies. Third, potential confounders such as dietary intake, physical activity, and inflammatory markers were not uniformly controlled across the studies. Fourth, the inclusion of different study designs (cross-sectional, cohort-derived, case-control, and RCT-derived data), as well as the possibility of partial overlap across publications from the same research groups despite efforts to avoid duplication, may have introduced methodological heterogeneity, which should be considered when the pooled estimates are interpreted. In addition, the limited number of studies included in the subgroup and meta-regression analyses may have reduced the ability to adequately explain the observed heterogeneity. Fifth, the search strategy was limited to major bibliographic databases and did not include gray literature sources (e.g., trial registries, conference proceedings, or dissertations), which may have introduced potential publication bias. Finally, publication bias and residual confounding factors cannot be fully excluded despite rigorous statistical adjustments. Moreover, variability in exposure and outcome assessment methods across studies, together with the transformation of heterogeneous effect measures into a common OR metric, may have introduced measurement heterogeneity and some degree of approximation, further limiting the comparability of results and the precision of the pooled estimates. Taken together, these limitations highlight that the current evidence base is primarily observational and cross-sectional, limiting the ability to draw causal inferences regarding the relationship between circulating carotenoids and vascular health.

Future studies should prioritize well-designed randomized controlled trials to establish causality between circulating carotenoids and vascular outcomes, ideally using standardized IMT measurements across diverse populations. Research should also explore the bioavailability and metabolism of carotenoids in relation to lifestyle and dietary patterns, as well as their interactions with traditional cardiovascular risk factors. In addition, sex-specific analyses are warranted to clarify the hormonal and metabolic influences on carotenoid-related vascular protection.

## 5. Conclusions

In conclusion, our findings suggest that lycopene, but not β-carotene, is consistently associated with lower carotid IMT, supporting its potential role in cardiovascular risk prevention. Clinically, promoting the consumption of lycopene-rich foods (e.g., tomatoes and red fruits) may contribute to vascular health. Future research should prioritize RCTs to establish causality, investigate the bioavailability of carotenoids across populations, and explore personalized antioxidant interventions, particularly those that consider sex-based metabolic and vascular differences.

## Figures and Tables

**Table 1:** Characteristics of the included studies in the systematic review for the association between antioxidants and intima–media thickness.

References	Country	Study Design	Population Characteristics			Exposure: Antioxidants		Outcome: IMT	
			Sample Size (n, %Female)	Mean Age (Years)	Comorbidities (%)	Type	Basal Plasma Levels (µmol/L)	Device	Basal Levels (mm)
Rissanen et al., 2000 [27]	Finland	Cross-sectional data from an RCT	520 (50.8)	59.8 ± 5.4	NA	Lycopene	NA	Biosound Phase 2	0.998 ± 0.258
Gale et al., 2001 [28]	United Kingdom	Cross-sectional data from a cohort study	468 (43.6)	70.2 ± 2.1	NA	β-Carotene	0.23 ± 2.3	HDI3000 high-resolution	0.828 ± 0.145
McQuillan et al., 2001 [29]	Australia	Cross-sectional data from a cohort study	1111 (49.8)	52.5 ± 13.0	HT (24.0) DM (1.2) HLP (24.6) MI (4.1) Stroke (1.2)	β-Carotene Lycopene	0.76 ± 0.67 0.40 ± 0.26	128 XP/10 mainframe	0.710 ± 0.140
Gianetti et al., 2002 [30]	Italy	Cross-sectional data from a case-control study	33 (18.2)	57.4 ± 8.8	HT (66.7)	β-Carotene Lycopene	0.97 ± 0.66 0.93 ± 0.49	AU 590 Asynchronous Scanner	1.620 ± 0.490
Rissanen et al., 2002 [31]	Finland	Cross-sectional data from an RCT	520 (50.8)	59.8 ± 5.4	NA	Lycopene	0.16 ± 0.11	Biosound Phase 2	1.000 ± 0.257
Rissanen et al., 2003 [32]	Finland	Cross-sectional data from a cohort study	1028 (0.0)	56.2 ± 6.3	NA	β-Carotene Lycopene	0.41 ± 0.28 0.15 ± 0.14	Biosound Phase 2	0.880 (0.860–0.905)
Riccioni et al., 2008 [33]	USA	Cross-sectional study	220 (50.5)	46.4 ± 17.8	Atherosclerosis (56.8)	β-Carotene Lycopene	2.14 ± 0.56 0.63 ± 0.31	Acuson Sequoia C512	NA
Riccioni et al., 2009 [34]	USA	Cross-sectional data from a cohort study	640 (53.6)	57.9 ± 11.5	HT (16.6) DM (1.3) HLP (16.4) MI (0.6)	β-Carotene	1.31 ± 0.39	Acuson Sequoia C512	NA
Karppi et al., 2011 [35]	Finland	Cross-sectional data from a cohort study	1212 (0.0)	71.6 ± 5.1	HT (65.5) DM (39.3) CHD (32.8)	β-Carotene Lycopene	0.45 ± 0.32 0.09 ± 0.06	Esaote Technos MP	NA
Riccioni et al., 2011 [36]	Italy	Cross-sectional data from a cohort study	120 (51.7)	51.3 ± 10.6	NA	Lycopene	NA	Acuson Sequoia C512	NA
Zou et al., 2011 [37]	China	Cross-sectional data from a cohort study	232 (68.1)	55.5 ± 5.4	HT (47.8) DM (15.1) HLP (63.8)	β-Carotene Lycopene	0.08 ± 0.01 0.10 ± 0.05	Aloka Prosound α-10	0.711 ± 0.153
Xu et al., 2012 [38]	China	Cross-sectional data from a case-control study	80 (62.5)	55.6 ± 5.1	DM (13.0) HLP (27.3)	β-Carotene Lycopene	0.08 ± 0.01 0.09 ± 0.04	Aloka Prosound α-10	NA
Wang et al., 2018 [39]	China	Cross-sectional study	2947 (68.3)	58.6 ± 6.2	NA	β-Carotene Lycopene	0.53 ± 0.38 0.19 ± 0.13	Aplio	0.704 ± 0.148

Data are shown as the mean ± standard deviation (SD) or interquartile range (IQ). CHD: coronary heart disease; DM: diabetes mellitus; HLP: hyperlipidemia; HT: hypertension; IMT: intima–media thickness; MI: myocardial infarction; NA: not available; RCT: randomized controlled trial.

## Data Availability

The original contributions presented in the study are included in the article, and further inquiries can be directed to the corresponding author.

## Supplementary Materials

The following supporting information can be downloaded at https://www.mdpi.com/article/10.3390/nu18071043/s1, Table S1. PRISMA 2020 checklist; Table S2. Search strategy for the PubMed database; Table S3. Original effect measures and harmonization approach for baseline associations between circulating lycopene and carotid intima–media thickness; Table S4. Original effect measures and harmonization approach for baseline associations between circulating β-carotene and carotid intima–media thickness; Table S5. Covariates of included studies in the systematic review for the association between antioxidants and intima media thickness; Table S6. Covariates included in multivariable models for baseline associations between circulating β-carotene and carotid intima–media thickness; Table S7. Quality assessment with the tool for controlled intervention studies of the National Heart, Lung and Blood Institute for the association between antioxidants and intima media thickness; Table S8. Quality assessment with the tool for case-control studies of the National Heart, Lung and Blood Institute for the association between antioxidants and intima media thickness; Table S9. Quality assessment with the tool for observational cohort and cross-sectional studies of the National Heart, Lung and Blood Institute for the association between antioxidants and intima media thickness; Table S10. Subgroup analysis according to gender (male or female) for the association between antioxidants and intima media thickness.; Table S11. Meta-regression models according to mean age, body mass index, systolic blood pressure, diastolic blood pressure, total cholesterol, HDL cholesterol, LDL cholesterol, triglycerides and current smoker for the association between antioxidants and intima media thickness; Figure S1. Assessment of publication bias by funnel plots for the association between between lycopene and intima media thickness; Figure S2. Assessment of publication bias by funnel plots for the association between β-carotene and intima media thickness.

## Abbreviations

The following abbreviations are used in this manuscript:

IMT	Intima–media thickness
PRISMA	Preferred Reporting Items for Systematic Reviews and Meta-analyses
PROSPERO	International Prospective Register of Systematic Reviews
OR	Odds ratio
CI	Confidence interval
CVDs	Cardiovascular diseases
LDL	Low-density lipoprotein
RCT	Randomized Controlled Trial
PICO	Population, Intervention, Comparator, Outcome
BMI	Body mass index
SBP	Systolic blood pressure
DBP	Diastolic blood pressure
HDL	High-density lipoprotein
ROS	Reactive oxygen species
*I*^2^	I-squared statistic (heterogeneity index)
STATA	Statistical software for data science
NHLBI	National Heart, Lung, and Blood Institute
