# Supplementary material for: Circulating Lycopene and β-Carotene Levels Are Inversely Associated with Carotid Intima–Media Thickness: A Systematic Review and Meta-Analysis
Source: Nutrients. 2026 Mar 25;18(7):1043. doi: 10.3390/nu18071043 (PMC13074833; doi:10.3390/nu18071043)

**Supplementary material**

**Table S1.** PRISMA 2020 checklist.

| Section and Topic | Item # | Checklist item | Location where item is reported |
|---|---|---|---|
| **TITLE** | | | |
| Title | 1 | Identify the report as a systematic review. | Title |
| **ABSTRACT** | | | |
| Abstract | 2 | See the PRISMA 2020 for Abstracts checklist. | Abstract |
| **INTRODUCTION** | | | |
| Rationale | 3 | Describe the rationale for the review in the context of existing knowledge. | Introduction (final paragraph) |
| Objectives | 4 | Provide an explicit statement of the objective(s) or question(s) the review addresses. | Introduction (last paragraph) |
| **METHODS** | | | |
| Eligibility criteria | 5 | Specify the inclusion and exclusion criteria for the review and how studies were grouped for the syntheses. | Section 2.2 |
| Information sources | 6 | Specify all databases, registers, websites, organisations, reference lists and other sources searched or consulted to identify studies. Specify the date when each source was last searched or consulted. | Section 2.1 |
| Search strategy | 7 | Present the full search strategies for all databases, registers and websites, including any filters and limits used. | Section 2.1; Table S1 |
| Selection process | 8 | Specify the methods used to decide whether a study met the inclusion criteria of the review, including how many reviewers screened each record and each report retrieved, whether they worked independently, and if applicable, details of automation tools used in the process. | Section 2 |
| Data collection process | 9 | Specify the methods used to collect data from reports, including how many reviewers collected data from each report, whether they worked independently, any processes for obtaining or confirming data from study investigators, and if applicable, details of automation tools used in the process. | Section 2.3 |
| Data items | 10a | List and define all outcomes for which data were sought. Specify whether all results that were compatible with each outcome domain in each study were sought (e.g. for all measures, time points, analyses), and if not, the methods used to decide which results to collect. | |

| Section and Topic | Item # | Checklist item | Location where item is reported |
|---|---|---|---|
| | | | Section 2.3 |
| | 10b | List and define all other variables for which data were sought (e.g. participant and intervention characteristics, funding sources). Describe any assumptions made about any missing or unclear information. | Section 2.3 |
| Study risk of bias assessment | 11 | Specify the methods used to assess risk of bias in the included studies, including details of the tool(s) used, how many reviewers assessed each study and whether they worked independently, and if applicable, details of automation tools used in the process. | Section 2.4; Supplementary Table |
| Effect measures | 12 | Specify for each outcome the effect measure(s) (e.g. risk ratio, mean difference) used in the synthesis or presentation of results. | Section 2.5 |
| Synthesis methods | 13a | Describe the processes used to decide which studies were eligible for each synthesis (e.g. tabulating the study intervention characteristics and comparing against the planned groups for each synthesis (item #5)). | Section 2.5 |
| | 13b | Describe any methods required to prepare the data for presentation or synthesis, such as handling of missing summary statistics, or data conversions. | Section 2.5 |
| | 13c | Describe any methods used to tabulate or visually display results of individual studies and syntheses. | Section 2.5 |
| | 13d | Describe any methods used to synthesize results and provide a rationale for the choice(s). If meta-analysis was performed, describe the model(s), method(s) to identify the presence and extent of statistical heterogeneity, and software package(s) used. | Section 2.5 |
| | 13e | Describe any methods used to explore possible causes of heterogeneity among study results (e.g. subgroup analysis, meta-regression). | Section 2.5 |
| | 13f | Describe any sensitivity analyses conducted to assess robustness of the synthesized results. | Section 2.5 |
| Reporting bias assessment | 14 | Describe any methods used to assess risk of bias due to missing results in a synthesis (arising from reporting biases). | Section 2.5; Discussion |
| Certainty assessment | 15 | Describe any methods used to assess certainty (or confidence) in the body of evidence for an outcome. | - |
| **RESULTS** | | | |
| Study selection | 16a | Describe the results of the search and selection process, from the number of records identified in the search to the number of studies | Figure 1 |

| Section and Topic | Item # | Checklist item | Location where item is reported |
|---|---|---|---|
| | | included in the review, ideally using a flow diagram. | |
| | 16b | Cite studies that might appear to meet the inclusion criteria, but which were excluded, and explain why they were excluded. | Figure 1 |
| Study characteristics | 17 | Cite each included study and present its characteristics. | Table 1 |
| Risk of bias in studies | 18 | Present assessments of risk of bias for each included study. | Supplementary + Figure 2 |
| Results of individual studies | 19 | For all outcomes, present, for each study: (a) summary statistics for each group (where appropriate) and (b) an effect estimate and its precision (e.g. confidence/credible interval), ideally using structured tables or plots. | - |
| Results of syntheses | 20a | For each synthesis, briefly summarise the characteristics and risk of bias among contributing studies. | Section 3.3 |
| | 20b | Present results of all statistical syntheses conducted. If meta-analysis was done, present for each the summary estimate and its precision (e.g. confidence/credible interval) and measures of statistical heterogeneity. If comparing groups, describe the direction of the effect. | Section 3.3 |
| | 20c | Present results of all investigations of possible causes of heterogeneity among study results. | Section 3.3 |
| | 20d | Present results of all sensitivity analyses conducted to assess the robustness of the synthesized results. | Section 3.3 |
| Reporting biases | 21 | Present assessments of risk of bias due to missing results (arising from reporting biases) for each synthesis assessed. | Results + Discussion |
| Certainty of evidence | 22 | Present assessments of certainty (or confidence) in the body of evidence for each outcome assessed. | - |
| **DISCUSSION** | | | |
| Discussion | 23a | Provide a general interpretation of the results in the context of other evidence. | Discussion section |
| | 23b | Discuss any limitations of the evidence included in the review. | Discussion section |
| | 23c | Discuss any limitations of the review processes used. | Discussion section |
| | 23d | Discuss implications of the results for practice, policy, and future research. | Discussion |

| Section and Topic | Item # | Checklist item | Location where item is reported |
|---|---|---|---|
| | | | section |
| **OTHER INFORMATION** | | | |
| Registration and protocol | 24a | Provide registration information for the review, including register name and registration number, or state that the review was not registered. | Discussion (limitations paragraph) |
| | 24b | Indicate where the review protocol can be accessed, or state that a protocol was not prepared. | Discussion (limitations paragraph) |
| | 24c | Describe and explain any amendments to information provided at registration or in the protocol. | Discussion (limitations paragraph) |
| Support | 25 | Describe sources of financial or non-financial support for the review, and the role of the funders or sponsors in the review. | Discussion (final paragraphs) |
| Competing interests | 26 | Declare any competing interests of review authors. | Page 7 |
| Availability of data, code and other materials | 27 | Report which of the following are publicly available and where they can be found: template data collection forms; data extracted from included studies; data used for all analyses; analytic code; any other materials used in the review. | Page 6 |

**Table S2.** Search strategy for the PubMed database.

| | |
|---|---|
| 1 antioxidant lycopene [All Fields]<br>2 lycopene [Supplementary Concept]<br>3 lycopene [All Fields]<br>4 lycopene [MeSH Terms]<br>5 plasma lycopene [All Fields]<br>6 serum lycopene [All Fields]<br>7 plasma carotenoids [All Fields]<br>8 serum carotenoid [All Fields]<br>9 carotenoids [Supplementary Concept]<br>10 carotenoids [All Fields]<br>11 carotenoids [MeSH Terms]<br>12 antioxidant vitamins [All Fields]<br>13 plasma antioxidants [All Fields]<br>14 antioxidants [All Fields]<br>15 antioxidants [Supplementary Concept]<br>16 antioxidants [MeSH Terms]<br>17 beta carotene [Supplementary Concept]<br>18 beta carotene [All Fields]<br>19 beta carotene [MeSH Terms]<br>20 alpha-carotene [Supplementary Concept]<br>21 alpha-carotene [All Fields]<br>22 alpha carotene [MeSH Terms]<br>23 beta-cryptoxanthin [Supplementary Concept]<br>24 beta-cryptoxanthin [All Fields]<br>25 beta cryptoxanthin [MeSH Terms]<br>26 lutein [Supplementary Concept] | 27 lutein [All Fields]<br>28 lutein [MeSH Terms])<br>29 zeaxanthins [Supplementary Concept] 30 zeaxanthins [All Fields]<br>31 zeaxanthin [All Fields]<br>32 zeaxanthins [MeSH Terms]<br>33 1 OR 2 OR 3 OR 4 OR 5 OR 6 OR 7 OR 8 OR 9 OR 10 OR 11 OR 12 OR 13 OR 14 OR 15 OR 16 OR 17 OR 18 OR 19 OR 20 OR 21 OR 22 OR 23 OR 24 OR 25 OR 26 OR 27 OR 28 OR 29 OR 30 OR 31 OR 32<br>34 carotid intima-media thickness [All Fields]<br>35 intima-media thickness [All Fields]<br>36 cimt [All Fields]<br>37 IMT [All Fields]<br>38 34 OR 35 OR 36 OR 37<br>39 33 AND 38<br>40 atherosclerosis [MeSH Terms]<br>41 atherosclerosis [All Fields])<br>42 carotid artery wall [All Fields]<br>43 carotid atherosclerosis [All Fields]<br>44 carotid atherosclerotic disease [All Fields]<br>45 carotid arteries [All Fields]<br>46 40 OR 41 OR 42 OR 43 OR 44 OR 45<br>47 39 AND 46 |

**Table S3.** Original effect measures and harmonization approach for baseline associations between circulating lycopene and carotid intima–media thickness

| **Reference** | **Exposure** | **Original effect measure** | **Exposure scale** | **Transformation method** |
|---|---|---|---|---|
| McQuillan et al., 2001 (a) | Lycopene | Mean IMT by quartiles | Quartiles (Q2–Q4 vs Q1) | Comparisons vs Q1; pooled estimate → SMD → OR |
| McQuillan et al., 2001 (b) | Lycopene | Mean IMT by quartiles | Quartiles (Q2–Q4 vs Q1) | Comparisons vs Q1; pooled estimate → SMD → OR |
| Gianetti et al., 2002 | Lycopene | Correlation coefficient (r) | Continuous | r → Fisher's z → OR |
| Rissanen et al., 2002 (a) | Lycopene | Correlation coefficient (r) | Continuous | r → Fisher's z → OR |
| Rissanen et al., 2002 (b) | Lycopene | Correlation coefficient (r) | Continuous | r → Fisher's z → OR |
| Karppi et al., 2011 | Lycopene | Correlation coefficient (r) | Continuous | r → Fisher's z → OR |
| Riccioni et al., 2011 | Lycopene | Odds ratio (OR) | Categorical | Inverse OR to align direction |
| Zou et al., 2011 | Lycopene | Correlation coefficient (r) | Continuous | r → Fisher's z → OR |
| Wang et al., 2018 | Lycopene | Mean IMT by quartiles | Quartiles (Q2–Q4 vs Q1) | Comparisons vs Q1; pooled estimate → SMD → OR |

IMT: intima media thickness; SMD: standardized mean difference; OR: odds ratio.

**Table S4.** Original effect measures and harmonization approach for baseline associations between circulating β-carotene and carotid intima–media thickness

| Reference | Exposure | Original effect measure | Exposure scale | Transformation method |
|---|---|---|---|---|
| McQuillan et al., 2001 (a) | β-carotene | Mean IMT by quartiles | Quartiles (Q2–Q4 vs Q1) | Comparisons vs Q1; pooled estimate → SMD → OR |
| McQuillan et al., 2001 (b) | β-carotene | Mean IMT by quartiles | Quartiles (Q2–Q4 vs Q1) | Comparisons vs Q1; pooled estimate → SMD → OR |
| Gale et al., 2002 (a) | β-carotene | Regression coefficient (β) | Continuous | β → standardized effect → OR |
| Gale et al., 2002 (b) | β-carotene | Regression coefficient (β) | Continuous | β → standardized effect → OR |
| Gianetti et al., 2002 | β-carotene | Correlation coefficient (r) | Continuous | r → Fisher's z → OR |
| Karppi et al., 2011 | β-carotene | Correlation coefficient (r) | Continuous | r → Fisher's z → OR |
| Zou et al., 2011 | β-carotene | Correlation coefficient (r) | Continuous | r → Fisher's z → OR |
| Wang et al., 2018 | β-carotene | Mean IMT by quartiles | Quartiles (Q2–Q4 vs Q1) | Comparisons vs Q1; pooled estimate → SMD → OR |

IMT: intima media thickness; SMD: standardized mean difference; OR: odds ratio.

**Table S5.** Covariates of included studies in the systematic review for the association between antioxidants and intima media thickness.

| Reference | BMI (kg/m2) | SBP (mmHg) | DBP (mmHg) | Total cholesterol (mmol/L) | HDL cholesterol (mmol/L) | LDL cholesterol (mmol/L) | Triglycerides (mmol/L) | Current smoker (%) |
|---|---|---|---|---|---|---|---|---|
| Rissanen et al. 2000 | 26.1 ± 3.1 | 133.3 ± 18.9 | 79.3 ± 8.9 | 6.4 ± 1.0 | 1.33 ± 0.3 | 4.5 ± 1.0 | 1.6 ± 0.8 | 47.7 |
| Gale et al. 2001 | 27.2 ± 4.6 | 143.5 ± 21.2 | 80.1 ± 10.0 | 6.2 ± 1.2 | 1.3 ± 1.3 | 4.2 ± 1.1 | NA | NA |
| McQuillan et al. 2001 | NA | 129.0 ± 19.0 | 81.0 ± 10.0 | 5.6 ± 1.0 | 1.4 ± 0.4 | 3.7 ± 0.9 | 1.3 ± 0.7 | 15.0 |
| Gianetti et al. 2002 | 26.0 ± 2.6 | NA | NA | 5.6 ± 0.8 | 1.2 ± 0.3 | 3.7 ± 0.9 | 1.5 (0.9 – 2.5) | 24.2 |
| Rissanen et al. 2002 | 26.1 ± 3.1 | 133.3 ± 25.2 | 79.3 ± 8.9 | 6.4 ± 1.0 | 1.3 ± 0.3 | 4.5 ± 1.0 | 1.6 ± 0.8 | 47.7 |
| Rissanen et al. 2003 | 27.4 ± 3.6 | 134.9 ± 16.5 | NA | 5.5 ± 0.9 | 1.1 ± 0.3 | 3.9 ± 0.9 | 1.6 ± 1.0 | NA |
| Riccioni et al. 2008 | 25.6 ± 3.1 | 127.0 ± 5.5 | 83.5 ± 5.5 | 4.9 ± 0.7 | 1.3 ± 0.1 | 3.2 ± 0.6 | 1.5 ± 0.4 | 49.5 |
| Riccioni et al. 2009 | 27.5 ± 2.5 | NA | NA | 5.0 ± 0.6 | 1.3 ± 0.1 | 3.8 ± 0.6 | 1.8 ± 0.3 | 12.2 |
| Karppi et al. 2011 | 27.0 ± 3.9 | 134.5 ± 17.3 | NA | 4.7 ± 1.0 | 1.1 ± 0.3 | 2.9 ± 0.8 | 1.2 ± 0.6 | 8.9 |
| Riccioni et al. 2011 | 25.3 ± 4.2 | NA | NA | NA | NA | NA | NA | 37.5 |
| Zou et al. 2011 | 25.0 ± 3.2 | 124.1 ± 15.0 | 79.8 ± 9.7 | 5.2 ± 1.0 | 1.4 ± 0.3 | 3.1 ± 0.8 | 1.9 ± 1.5 | 24.3 |
| Xu et al. 2012 | 24.7 ± 2.7 | 123.9 ± 15.7 | 79.2 ± 9.2 | 5.1 ± 0.9 | 1.4 ± 1.3 | 3.1 ± 0.7 | 1.8 ± 1.2 | 22.1 |
| Wang et al. 2018 | 23.3 ± 3.1 | 124.1 ± 17.7 | 77.4 ± 10.6 | 5.5 ± 1.1 | 1.4 ± 0.3 | 3.6 ± 0.9 | 1.6 ± 1.3 | 16.0 |

Data are shown as mean±standard deviation (SD) or interquartile range (IQ). BMI: body mass index; DBP: diastolic blood pressure; HDL: high density lipoprotein; LDL: low density lipoprotein; NA: not available; SBP: systolic blood pressure.

**Table S6.** Covariates included in multivariable models for baseline associations between circulating β-carotene and carotid intima–media thickness.

| Reference | Age | Sex | BMI | Smoking | Blood pressure | Lipid profile | Other covariates | Adjustment level |
|---|---|---|---|---|---|---|---|---|
| McQuillan et al. 2001 | ✓ | NR | NR | ✓ | ✓ (SBP) | ✓ (LDL) | Homocysteine, diabetes, family history, total energy intake | Fully adjusted |
| Gale et al. 2002 | ✓ | NR | NR | ✓ | ✓ (pulse pressure / SBP) | ✓ (total cholesterol, LDL) | Hypertension, alcohol intake | Fully adjusted |
| Gianetti et al. 2002 | NR | NR | NR | NR | NR | NR | — | Unadjusted |
| Rissanen et al. 2002 | NR | NR | NR | NR | NR | NR | — | Unadjusted |
| Karppi et al. 2011 | NR | NR | NR | NR | NR | NR | — | Unadjusted |
| Riccioni et al. 2011 | NR | NR | NR | NR | NR | ✓ (total cholesterol, LDL, triglycerides) | — | Partially adjusted |
| Zou et al. 2011 | ✓ | ✓ | NR | NR | NR | NR | — | Partially adjusted |
| Wang et al. 2018 | ✓ | ✓ | ✓ | ✓ | NR | ✓ (dietary fat, cholesterol) | Education, income, alcohol, physical activity, dietary factors (energy, fat, fibre) | Fully adjusted |

BMI: body mass index; NR: not reported; SBP: systolic blood pressure; LDL:low density lipoprotein

**Table S7.** Quality assessment with the tool for controlled intervention studies of the National Heart, Lung and Blood Institute for the association between antioxidants and intima media thickness.

| Reference | 1 | 2 | 3 | 4 | 5 | 6 | 7 | 8 | 9 | 10 | 11 | 12 | 13 | 14 | Quality |
|---|---|---|---|---|---|---|---|---|---|---|---|---|---|---|---|
| Rissanen et al. 2000 | Y | NR | Y | Y | N | Y | NR | NR | NR | Y | Y | N | Y | Y | Fair |
| Rissanen et al. 2002 | Y | NR | Y | Y | N | Y | NR | NR | NR | Y | Y | N | Y | Y | Fair |

N: no; NA, not applicable; NR: not reported; Y: yes.

**Table S8.** Quality assessment with the tool for case-control studies of the National Heart, Lung and Blood Institute for the association between antioxidants and intima media thickness.

| Reference | 1 | 2 | 3 | 4 | 5 | 6 | 7 | 8 | 9 | 10 | 11 | 12 | Quality |
|---|---|---|---|---|---|---|---|---|---|---|---|---|---|
| Gianetti et al. 2002 | Y | Y | N | Y | Y | Y | NR | NR | Y | Y | Y | N | Good |
| Xu et al. 2012 | Y | Y | N | Y | Y | Y | NR | Y | Y | Y | N | Y | Good |

N: no; NA, not applicable; NR: not reported; Y: yes.

**Table S9.** Quality assessment with the tool for observational cohort and cross-sectional studies of the National Heart, Lung and Blood Institute for the association between antioxidants and intima media thickness.

| Reference | 1 | 2 | 3 | 4 | 5 | 6 | 7 | 8 | 9 | 10 | 11 | 12 | 13 | 14 | Quality |
|---|---|---|---|---|---|---|---|---|---|---|---|---|---|---|---|
| Gale et al. 2001 | Y | Y | Y | Y | N | Y | NR | Y | Y | N | Y | NR | Y | Y | Good |
| McQuillan et al. 2001 | Y | Y | Y | Y | N | Y | Y | Y | Y | N | Y | NR | NR | Y | Good |
| Rissanen et al. 2003 | Y | Y | NR | Y | N | Y | NR | Y | Y | N | Y | NR | N | Y | Fair |
| Riccioni et al. 2008 | Y | Y | NR | Y | N | Y | NA | Y | Y | NA | Y | Y | NA | N | Good |
| Riccioni et al. 2009 | Y | Y | NR | Y | N | Y | NR | Y | Y | N | Y | Y | NR | Y | Fair |
| Karppi et al. 2011 | Y | Y | NR | Y | N | Y | NR | Y | Y | N | Y | NR | N | Y | Fair |
| Riccioni et al. 2011 | Y | Y | NR | Y | N | Y | NR | Y | Y | N | Y | NR | NR | Y | Fair |
| Zou et al. 2011 | Y | Y | NR | Y | Y | Y | NR | Y | Y | N | Y | NR | Y | Y | Good |
| Wang et al. 2018 | Y | Y | Y | N | N | Y | NA | Y | Y | NA | Y | Y | NA | Y | Good |

N: no; NA, not applicable; NR: not reported; Y: yes.

**Table S10.** Subgroup analysis according to gender (male or female) for the association between antioxidants and intima media thickness.

| | n studies | OR (95%ICs) | $I^2$ |
|---|---|---|---|
| **Lycopene** | | | |
| Males | **3** | **0.61 (0.44, 0.86)** | **72.8%** |
| Females | **2** | **0.74 (0.58, 0.95)** | **0.0%** |
| **β-carotene** | | | |
| Males | 3 | 0.95 (0.88, 1.03) | 21.7% |
| Females | 2 | 0.99 (0.96, 1.01) | 0.0% |

OR: odds ratio

**Table S11.** Meta-regression models according to mean age, body mass index, systolic blood pressure, diastolic blood pressure, total cholesterol, HDL cholesterol, LDL cholesterol, triglycerides and current smoker for the association between antioxidants and intima media thickness.

| | Coefficient | 95% CIs | p value |
|---|---|---|---|
| **Lycopene** | | | |
| Mean age | -0.003 | -0.046, 0.041 | 0.886 |
| BMI | -0.130 | -0.405, 0.145 | 0.259 |
| SBP | **-0.039** | **-0.070, -0.008** | **0.024** |
| DBP | 0.002 | -0.305, 0.310 | 0.982 |
| Total cholesterol | -0.167 | -0.739, 0.406 | 0.488 |
| HDL cholesterol | 0.712 | -0.983, 2.407 | 0.330 |
| LDL cholesterol | -0.211 | -0.782, 0.360 | 0.385 |
| Triglycerides | 0.372 | -0.575, 1.320 | 0.359 |
| Current smoker | -0.014 | -0.035, 0.006 | 0.133 |
| **β-carotene** | | | |
| Mean age | 0.003 | -0.000, 0.006 | 0.051 |
| BMI | **0.033** | **0.002, 0.065** | **0.043** |
| SBP | 0.005 | -0.005, 0.0152 | 0.262 |
| DBP | 0.022 | -0.059, 0.103 | 0.497 |
| Total cholesterol | 0.066 | -0.010, 0.142 | 0.077 |
| HDL cholesterol | 0.012 | -0.904, 0.929 | 0.975 |
| LDL cholesterol | **0.085** | **0.015, 0.155** | **0.026** |
| Triglycerides | 0.240 | -0.670, 1.151 | 0.504 |
| Current smoker | 0.001 | -0.001, 0.003 | 0.218 |

BMI: body mass index; DBP: diastolic blood pressure; HDL: high density lipoprotein; LDL: low density lipoprotein; SBP: systolic blood pressure.

**Figure S1.** Assessment of publication bias by funnel plots for the association between between lycopene and intima media thickness.

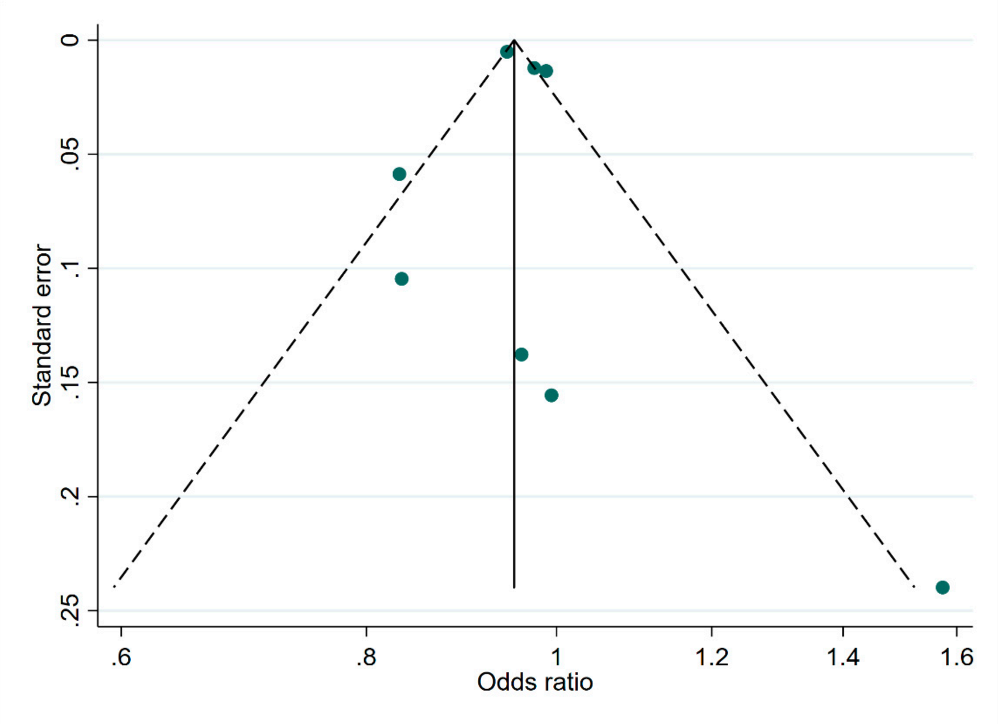


**Figure S2.** Assessment of publication bias by funnel plots for the association between β-carotene and intima media thickness.

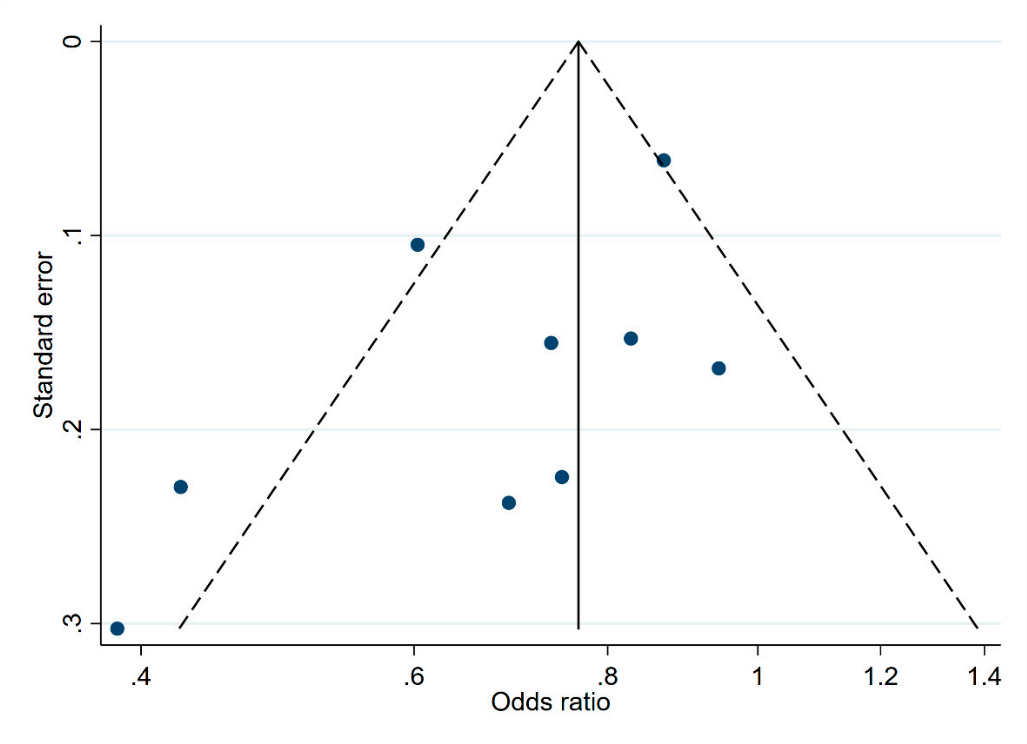

Supplement: Supplementary file 1 [file nutrients-18-01043-s001.zip › nutrients-4215456-supplementary.pdf]
